# Verbally Induced Olfactory Illusions Are Not Caused by Visual Processing: Evidence From Early and Late Blindness

**DOI:** 10.1177/20416695211016483

**Published:** 2021-05-22

**Authors:** Stina Cornell Kärnekull*, Billy Gerdfeldter, Maria Larsson, Artin Arshamian

**Affiliations:** Gösta Ekman Laboratory, Department of Psychology, 7675 Stockholm University, Stockholm, Sweden; Gösta Ekman Laboratory, Department of Psychology, 7675 Stockholm University, Stockholm, Sweden; Division of Psychology, Department of Clinical Neuroscience, Karolinska Institutet, Stockholm, Sweden

**Keywords:** blindness, odor pleasantness, olfactory illusion, verbal label, mental imagery

## Abstract

Olfactory perception is malleable and easily modulated by top-down processes such as those induced by visual and verbal information. A classic example of this is olfactory illusions where the perceived pleasantness of an odor is manipulated by the valence of a verbal label that is either visually or auditorily presented together with the odor. The mechanism behind this illusion is still unknown, and it is not clear if it is driven only by verbal information or if there is an interaction between language functions and visual mental imagery processes. One way to test this directly is to study early blind individuals who have little or no experience of visual information or visual mental imagery. Here, we did this by testing early blind, late blind, and sighted individuals in a classical paradigm where odors were presented with negative, neutral, and positive labels via speech. In contrast to our hypothesis—that the lack of visual imagery would render early blind individuals less susceptible to the olfactory illusion—early and late blind participants showed more amplified illusions than sighted. These findings demonstrate that the general mechanism underlying verbally induced olfactory illusions is not caused by visual processing and visual mental imagery per se.

## Introduction

Perception in all senses can be shaped by contextual factors and top-down processes, such as the interaction of multiple sensory modalities and language (Herz & von Clef, 2001; Majid & Levinson, 2011; Seo et al., 2010; Shams et al., 2000; Vroomen & Keetels, 2010). For example, the simultaneous presentation of several auditory beeps with a visual flash induces illusions of multiple flashes (Shams et al., 2000), and the well-known ventriloquism effect is observed when discrepant visual information biases the localization of simultaneously presented sounds (Bertelson & De Gelder, 2004). Similarly, top-down effects of language can affect perception as illustrated when color names warp the color space differently depending on the language the perceiver is speaking (Lupyan et al., 2020; Mitterer et al., 2009). The malleability of perception is especially evident in olfactory perception where decades of research demonstrate that the perception of odors is easily modulated by visual and verbal information (Herz & von Clef, 2001; Jadauji et al., 2012; Morrot et al., 2001; Stevenson, 2011). For example, Zellner and Kautz (1990) found that the odor intensity of colored solutions was perceived as stronger than identical solutions without color. Olfactory-visual integration has also been shown to affect odor detection and discrimination. For example, it has been shown that odor detection is facilitated by the simultaneous presentation of semantically congruent pictures (Gottfried & Dolan, 2003) and repetitive transcranial magnetic stimulation of the visual cortex improves odor quality discrimination (Jadauji et al., 2012). Furthermore, evidence from multisensory illusions demonstrates that visual information has the power to shift the quality of the odor percept. In a classical study by Morrot et al. (2001), red coloring of a white wine made wine students use olfactory descriptors typical for red wine rather than white when describing its quality. The authors suggested that color influences odor perception and verbalization through the formation of mental visual images of odor sources. Thus, visual information, such as color, may be associated with mental imagery and semantic knowledge and thereby prime odor perception and identification. It is therefore not surprising that semantic information itself can influence odor perception in a more direct way. Several studies have demonstrated that having access to the actual identity of an odor may affect how odors are perceived, not the least in terms of the odorants’ hedonic value (Bensafi et al., 2007; Distel & Hudson, 2001; Ferdenzi et al., 2017; Herz, 2003; Poncelet et al., 2010). For example, Distel and Hudson (2001) showed that the presence as compared with absence of odor names increased pleasantness, intensity, and familiarity ratings of odors.

However, verbalization of odors can have far more dramatic effects in the form of olfactory illusions if the identity of the odor source is ambiguous. Specifically, an ambiguous odor will be perceived as more pleasant if it is presented with a positive rather than a negative label (de Araujo et al., 2005; Djordjevic et al., 2008; Herz & von Clef, 2001; Manescu et al., 2014). In their seminal study, Herz and von Clef (2001) presented participants with five ambiguous odors on two separate occasions, with either a positive or negative label, for example, a mix of isovaleric and butyric acids with the label parmesan cheese or vomit. Olfactory illusions were primarily reflected in altered pleasantness ratings (e.g., increased pleasantness for parmesan as compared with vomit), but intensity and familiarity ratings were also affected albeit to a lesser extent (e.g., the vomit label increased the intensity while decreasing the familiarity of the odorant). These types of verbally induced olfactory illusions have later been replicated and extended in subsequent studies (de Araujo et al., 2005; Djordjevic et al., 2008; Herz & von Clef, 2001; Manescu et al., 2014). For example, Djordjevic et al. (2008) conducted a study where the number of experimental odors was considerably larger and where participants were presented with negative, positive, and neutral labels on a single occasion. In line with Herz and von Clef (2001), positive labels resulted in higher pleasantness ratings and lower intensity ratings than did negative labels. Moreover, intensity ratings were higher for negative labels than positive and neutral labels.

Together, these research findings demonstrate that odor perception is susceptible to and modulated by both visual and verbal information. But how do these two modulators of odor perception relate to each other? For example, if verbal labels such as vomit and parmesan induce visual mental images associated with these labels, then visual information, albeit in the form of mental images, could be the underlying driver for the odor illusions and not verbalization per se. Thus, to understand the mechanisms that underlie these illusions, it is necessary to separate the effect of visual mental imagery from that of verbalization. One direct way of testing this is to study the differences in olfactory illusions between sighted and blind individuals, in particular those who are congenitally blind or blind since an early age (i.e., early blind). If the association between language and odor perception is independent of vision, then early blind individuals should be as easily affected by odor labels as sighted, in terms of perceptual ratings. In contrast, if vision plays a significant role in the effect of language on perception, early blind individuals should be less susceptible to the illusion. Late blind individuals constitute an interesting comparison group to the early blind and sighted individuals as they are blind at present but at the same time have access to visual memories and an intact visual imagery capacity. If visual processing is essential, then the late blind group should be as susceptible to the illusion as sighted individuals. On the other hand, identification and verbalization of odors are dramatically different compared with identification of stimuli from other sensory modalities. Specifically, people, at least from the Western world, are remarkably poor at identifying odors when contextual cues are absent (Majid & Burenhult, 2014; Olofsson & Gottfried, 2015). Given this, and the fact that blind individuals have to rely more upon verbal descriptions in everyday life than sighted, one could argue that blind individuals should have stronger and not weaker olfactory illusions. In this case, a verbal label would have higher contextual value for a congenitally blind individual than for a sighted person who frequently judges odors by the visual context they perceive them in (Lundström et al., 2019).

However, verbalization and visual mental imagery may not be the only possible drivers. Perceptual illusions in other sensory modalities may give a clue to other mechanisms by which blindness may affect this well-known multimodal olfactory illusion. Specifically, it has been demonstrated that congenital or an early onset of blindness directly enhances both auditory and tactile skills (Hötting & Röder, 2004, 2009; Occelli et al., 2013). This enhancement makes early blind individuals less prone to integrate multisensory information which in turn also renders them to be less susceptible to multisensory illusions that involve audition and touch. The reason for keeping the sensory streams from the two modalities separated is that it enhances perceptual skills in one sensory modality to catch subtle differences (e.g., increase pitch discrimination). For example, congenitally blind individuals show a reduced audio-tactile ventriloquist effect (Occelli et al., 2012) and have generally weaker cross-modal illusions for audio-tactile stimuli (Hötting & Röder, 2004, 2009; Occelli et al., 2013). These findings suggest that congenital blindness may increase the ability to attend to a perceptual task and ignore distracting information from another modality. In short, the majority of evidence suggests that congenitally or early blind individuals are generally less affected by multisensory illusions. However, in contrast to audition and touch, the majority of studies in olfaction show small or, in most of the cases, no differences in olfactory abilities between sighted and blind people (e.g., odor threshold, discrimination, identification, and memory), with the only difference being in odor imagery where blind individuals outperform sighted (Cornell Kärnekull et al., 2016, 2018, 2020; Sorokowska et al., 2019). This suggests that blind individuals would not be able to draw on enhanced odors skills to separate verbal information from the odor percept per se.

In this preregistered study, we tested olfactory illusions in early blind, late blind, and sighted individuals. We hypothesized that a part of the illusion depends on verbal labels evoking visual mental images of the *odor source* which in turn modulates the olfactory percept. Specifically, we predicted that this would result in early blind participants being less susceptible to olfactory illusions or at least that the effect of odor label on perceived pleasantness and intensity should be smaller than for sighted and late blind individuals. We predicted that late blind and sighted participants would not differ in these ratings.

Moreover, we hypothesized that irrespective of visual status, odors with negative labels should be rated as less pleasant and more intense than odors with neutral and positive labels, and odors with neutral labels should be rated as less pleasant and intense than those with positive labels. We used an exploratory approach for investigating potential effects of odor label and blindness on familiarity ratings due to sparse previous research. As odor identification ability may potentially affect the degree of the olfactory illusion, we also included an odor identification test. However, as the majority of studies show that blindness does not result in enhanced olfactory skills, we did not expect any group differences in odor identification. Finally, to control for potential effects of demand characteristics, we analyzed the data as a function of the participants’ level of understanding of the study rationale.

## Methods

### Study Design

A mixed factorial design was used in which participants with different visual status (early blind, late blind, and sighted) were presented with odor labels of different valence (negative, neutral, and positive) in a pseudo-random order.

### Participants

Thirty-two blind (17 early blind and 15 late blind) and 32 sighted participants matched on sex and age participated (±3 years, with the exception of one participant-pair with approximately 4 years age difference). It is hard to estimate the effect size of how blindness would affect this illusion as there is no previous research on this subject but based on the pleasantness ratings presented in Djordjevic et al.’s (2008) study, we would require nine participants in each of the three groups (early blind, late blind, and sighted participants) and three measurements (negative, neutral, and positive labels) to reach 80% power for the main effect of measurement. Considering that early blind individuals are very rare and given the large effects presented in Djordjevic et al.’s study, we estimated that the present number of participants was a reasonable trade-off and enough to also capture an interaction effect on our main variable (pleasantness) of interest. The power analysis was done in G*Power (Faul et al., 2009). We used the same definition of blindness as the World Health Organization, that is, visual acuity below 0.05 (ICD-10, World Health Organization). A total of 17 participants were totally blind (11 early blind and 6 late blind), and the remaining blind participants had different levels of residual vision below 0.05. Blind participants who had become blind before the age of 3 years were categorized as early blind, whereas those who had become blind after the age of 3 years were categorized as late blind. Among the early blind, 13 were congenitally blind. Table 1 presents participant characteristics regarding age and sex distribution among the three study groups.

**Table 1.** Total Number of Participants, Number of Women and Men, and Mean, Standard Deviation, and Range of Age Are Presented as a Function of Visual Status (Early Blind, Late Blind, and Sighted).

	Early blind	Late blind	Sighted
*N* (women/men)	17 (6/11)	15 (4/11)	32 (10/22)
*M* age	57.1	55.8	56.7
*SD* age	11.1	13.2	12.0
*Range* age	29–69	26–73	26–72

For all participants, the inclusion criterion was 18 to 75 years of age and exclusion criteria were neurological disorders and anosmia or any other olfactory disorder affecting the sense of smell. For sighted participants, severe visual impairment and eye disease were additional exclusion criteria, whereas for the blind, visual acuity above 0.05 was an additional exclusion criterion. Blind individuals were recruited through advertisements at organizations and newsletters for people who are blind or visually impaired, and sighted individuals were recruited through convenience sampling and advertisements at notice boards and an online billboard (www.studentkaninen.se).

The Regional Ethical Review Board in Stockholm approved the study (2015/369-31/4) and all participants gave written or oral informed consent before participation. They were compensated with gift vouchers worth SEK 300 for their participation in the study, and blind participants were reimbursed for travel expenses. The study was preregistered at the Open Science Framework (https://osf.io/ptwzx).

### Materials

Experimental odors (*n* = 10) and distractor odors (*n* = 15) were selected based on previous studies (Djordjevic et al., 2008; Herz & von Clef, 2001; Manescu et al., 2014). Odors were presented in nontranslucent glass jars covered with a cotton pad. The odors were continuously evaluated by the experimenters and remade when the quality deviated from the original odor. Each experimental odor was presented 3 times and had corresponding negative, neutral, and positive labels, whereas the distractor odors were presented only once and had a negative, neutral, or positive label. The labels were recorded by one of the experimenters and played through a loudspeaker to keep the valence and intonation of the presented odor labels in the procedure equal for all participants. Table 2 presents the odors and odor labels.

**Table 2.** Experimental and Distractor Odors With Corresponding Odor Labels.

Odor	Negative label	Neutral label	Positive label
*Experimental odors*			
Parmesan cheese (Par)^a^^,c^	Dry vomit	Thirty-two	Cheese
Indole (Ind)^a^	Feces	Forty-four	Farm
Cumin oil (Cum)^a^^,c^	Old sneakers	Twenty-five	Indian food
Juniperberry (Jun)^a^^,c^	Disinfectant	Twenty-one	Unripe mango
Pine needle oil (Pin)^a^^,b,c^	Old turpentine	Thirty-one	Pine needles
Isoamyl acetate (IAA)^a^	Paint thinner	Thirty-five	Ripe banana
Geraniol (Ger)^a^^,c^	Cheap perfume	Thirty-nine	Geraniums
Almond oil (Alm)^a^	Glue	Fifty-one	Almond oil
Citral (Cit)^a^	Toilet cleaning detergent	Twenty-six	Squeezed lemons
Eugenol (Eug)^a^	Dentist’s office	Twenty-eight	Dried cloves
*Distractor odors*			
Isovaleric acid^a^	Old socks		
Butyric acid^a^	Rotten meat		
Phenetole	Car tire		
Fish	Rotten fish		
4-Phenylbutyric acid	Sour dishcloth		
Ylang ylang^a^		Twenty-seven	
Celery seed oil^a^		Twenty-four	
Honey essential oil		Thirty-three	
Thyme oil		Forty-five	
Violet oil		Fifty-eight	
Orange^a^			Orange peel
Jasmine^a^			Jasmine tea
Grapefruit oil^a^			Grapefruit juice
Peppermint oil^a^			Spearmint gum
Peach oil			Peach

*Note.* Stimuli were identical or similar to:

^a^Djordjevic et al. (2008);

^b^Herz & von Clef (2001); and

^c^Manescu et al. (2014).

### Procedure

Participants were asked not to wear any perfume or other perfumed products on the day of the testing and to not eat or drink anything else than water at least 30 minutes before the study. They were informed that the aim was to investigate whether vision may affect how odors are perceived and that the study consisted of two parts: odor perception and an odor identification test. In the first part, they would smell a number of odors and rate intensity, pleasantness, and familiarity. Each odor would be preceded by the label of the odor or a number. The experimenter explained that the labels were recorded and would be played through a loudspeaker as there were two experimenters conducting the study and everything should be kept equal. The experimenter emphasized that only the odors, irrespective of the labels/numbers, should be rated. Participants were further informed that in the second part of the study participants’ odor identification ability would be tested. They were asked to wear a blindfold during both experiments. The whole study took approximately 1 hour and 30 minutes.

#### Part 1

The protocol was adapted from Djordjevic et al. (2008). Ten experimental odors were presented 3 times each, following a negative, neutral, or positive label, respectively. To minimize the risk that participants would notice that experimental odors were repeated, they were interspersed with distractor odors. The presentations were interspersed with 15 distractor odors and presented in a pseudo-random order in which there were always at least 5 odors before the same experimental odor was repeated. This ensured that there was enough time and odors between each experiment odor as to minimize recognition. Moreover, there were at the most three odor labels of the same valence in a row. This was to minimize the possibility that the participants would pay attention to the valence of the labels per se. The distractor odors were presented following a negative (*n* = 5), neutral (*n* = 5), or positive label (*n* = 5). In total, there were 45 odor presentations, and the presentation order was uniquely randomized across the matched pairs of blind and sighted participants. The odors were presented for 3 seconds, and the interstimulus interval was 60 seconds. To minimize habituation, the participants took a 5-minutes break halfway through the experiment (i.e., after 23 trials).

Participants verbally rated the pleasantness, intensity, and familiarity of the odorants on a 9-point Likert-type scale that was presented by speech and ranged from 1 to 9 (1 = *very unpleasant*, 5 = *neutral*, 9 = *very pleasant*), *intensity* (1 = *not perceptible*, 9 = *very intense*) and *familiarity* (1 = *completely unfamiliar*, 9 = *very familiar*) of the odors.

#### Part 2

Odor identification was tested using the Sniffin’ Sticks (Sniffin’ Sticks Identification Test Plus 16, purple, Burghardt®, Wedel, Germany; Hummel et al., 1997). In this test, participants were presented with 16 odors, with a 45 seconds interstimulus interval and asked to identify the odor by selecting one of the four label alternatives that were read aloud by the experimenter (i.e., four-alternative forced choice). Identification performance was defined as proportion correct responses.

At the end of the experiment, participants were asked about their understanding of the study rationale.

### Data Analysis

In line with the preregistration, we performed separate mixed analyses of variance (ANOVAs) for pleasantness, intensity, and familiarity ratings using visual status (early blind, late blind, and sighted) as the between-subjects factor and odor label (negative, neutral, and positive) as the within-subjects factor. We also did appropriate follow-up analyses based on participants’ level of understanding of the study rationale. We used Cohen’s *d* as our measurement of effect size and considered 0.2 to be small, 0.5 medium, and 0.8 as large effects. Furthermore, pleasantness and familiarity ratings were correlated, with the hypothesis that all groups should demonstrate positive correlations. Finally, a one-way ANOVA was conducted on proportion correct odor identification with visual status (early blind, late blind, and sighted) as the between-subjects factor.

## Results

### Olfactory Illusions

#### Pleasantness Ratings

**Figure 1.**
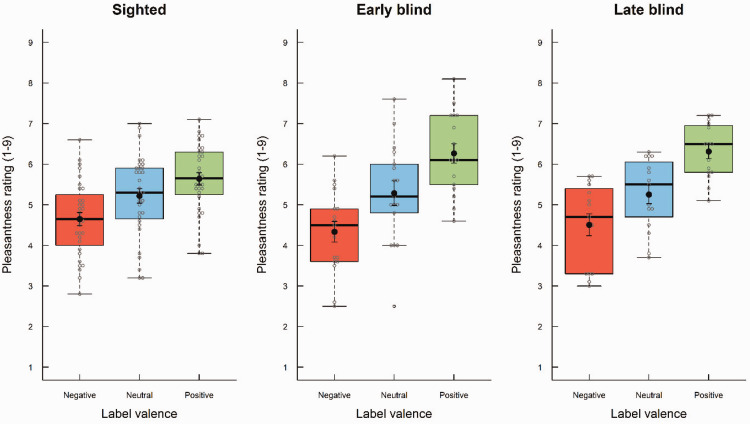
Boxplots of pleasantness ratings as a function of visual status (early blind, late blind, and sighted) and odor label (negative, neutral, and positive). The boxes indicate the 25th, 50th (median), and 75th percentiles of the distribution (lower, middle, and upper horizontal lines of the box). The upper hinges indicate the maximum value of the variable located within a distance of 1.5 times the interquartile range above the 75th percentile. The lower hinges indicate the corresponding distance to the 25th percentile value. Circles indicate values outside these hinges (outliers). The means are superimposed on the boxplots (filled circles).

Odor label influenced pleasantness ratings (Figure 1), *F*(2, 122) = 94.38, *p* < .001, η^2^ = .29, such that odors with positive labels (*M* = 5.96, standard deviation [*SD*] = 0.90) were rated as more pleasant than those with neutral (*M* = 5.25, *SD* = 1.04) and negative labels (*M* = 4.53, *SD* = 0.97), and those with neutral labels were rated as more pleasant than those with negative labels (*p*s < 0.001). Odor label interacted with visual status, *F*(4, 122) = 4.36, *p* = .002, η^2^ = .03, such that the effect of odor label on perceived pleasantness was larger for early and late blind participants than for the sighted, both in terms of the difference between positive and negative labels (Cohen’s *d*s = 1.12 and 1.17, respectively, indicated large effects) and between positive and neutral labels (Cohen’s *d*s = 0.80 and 1.11, respectively, indicated large effects; *p*s < .05). The group differences for the difference between neutral and negative labels were statistically nonsignificant. There was no overall difference between early blind (*M* = 5.30, *SD* = 0.91), late blind (*M* = 5.36, *SD* = 0.69), and sighted participants (*M* = 5.17, *SD* = 0.83) in their ratings, *F*(2, 61) = 0.31, *p* = .73, η^2^ = .005.

#### Intensity Ratings

**Figure 2.**
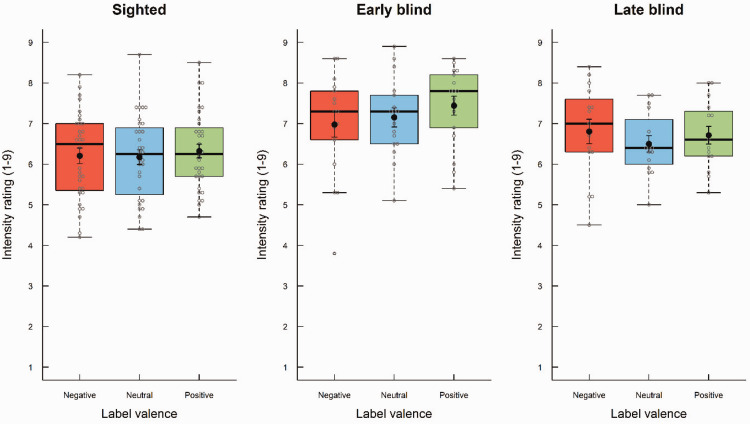
Boxplots of intensity ratings as a function of visual status (early blind, late blind, and sighted) and odor label (negative, neutral, and positive). The boxes indicate the 25th, 50th (median), and 75th percentiles of the distribution (lower, middle, and upper horizontal lines of the box). The upper hinges indicate the maximum value of the variable located within a distance of 1.5 times the interquartile range above the 75th percentile. The lower hinges indicate the corresponding distance to the 25th percentile value. Circles indicate values outside these hinges (outliers). The means are superimposed on the boxplots (filled circles).

There was an effect of odor label on intensity ratings (Figure 2), *F*(2, 122) = 4.03, *p* = .02, η^2^ = .007, as illustrated by slightly higher intensity ratings for odors with positive labels (*M* = 6.71, *SD* = 1.03) than those with neutral (*M* = 6.51, *SD* = 1.03) and negative labels (*M* = 6.55, *SD* = 1.19). Follow-up pairwise comparisons showed that only the difference between positive and neutral labels was statistically significant (*p* < .05). There was a small but statistically nonsignificant interaction between odor label and visual status, with larger effect of odor label for early and late blind participants, *F*(4, 122) = 2.14, *p* = .08, η^2^ = .007. Overall, early blind participants reported higher odor intensity (*M* = 7.19, *SD* = 0.99) than late blind (*M* = 6.67, *SD* = 0.88) and sighted participants (*M* = 6.24, *SD* = 0.97), *F*(2, 61) = 5.58, *p* = .006, η^2^ = .14. Post hoc tests showed that only the difference between early blind and sighted participants was statistically significant (*p* < .05).

#### Familiarity Ratings

**Figure 3.**
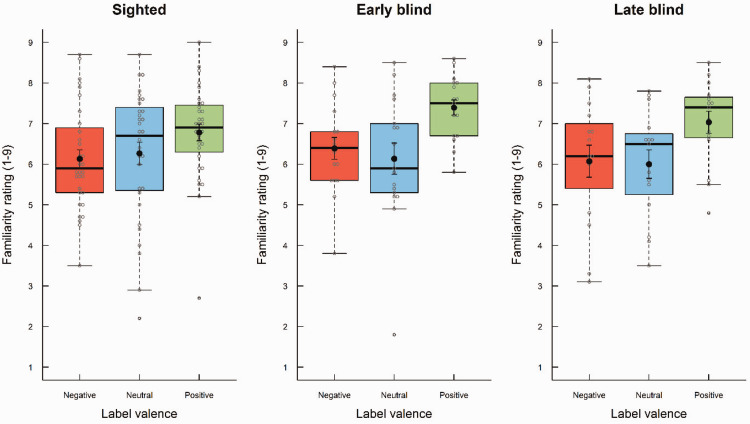
Boxplots of familiarity ratings as a function of visual status (early blind, late blind, and sighted) and odor label (negative, neutral, and positive). The boxes indicate the 25th, 50th (median), and 75th percentiles of the distribution (lower, middle, and upper horizontal lines of the box). The upper hinges indicate the maximum value of the variable located within a distance of 1.5 times the interquartile range above the 75th percentile. The lower hinges indicate the corresponding distance to the 25th percentile value. Circles indicate values outside these hinges (outliers). The means are superimposed on the boxplots (filled circles).

As the assumption of sphericity was violated in the ANOVA, the Greenhouse–Geisser correction was used. Odor label influenced familiarity ratings (Figure 3), *F*(1.80, 109.75) = 26.28, *p* < .001, η^2^ = .09, such that odors with positive labels (*M* = 7.00, *SD* = 1.06) were perceived as more familiar than those with neutral (*M* = 6.17, *SD* = 1.52) and negative labels (*M* = 6.19, *SD* = 1.28). Follow-up pairwise comparisons showed that only the difference between positive and negative labels was statistically significant (*p* < .05). The effect of odor label did not interact with visual status, *F*(3.598, 109.754) = 1.46, *p* = .225, η^2^ = .01, and overall familiarity ratings were similar for early blind (*M* = 6.64, *SD* = 0.98), late blind (*M* = 6.37, *SD* = 1.16), and sighted participants (*M* = 6.39, *SD* = 1.22), *F*(2, 61) = 0.31, *p* = .736, η^2^ = .007.

#### Follow-Up Analyses: Demand Characteristics

Based on participants’ responses to the question of their understanding of the rationale of the study, participants were categorized into three groups in accordance with Djordjevic et al. (2008): no understanding, partial understanding, and full understanding. Two of the authors (S. C. K. and B. G.) categorized the participants independently and the interrater reliability was high (*r* = .92). Cases of disagreement were discussed and resolved together. Thirty participants (5 early blind, 9 late blind, and 16 sighted) were categorized as having no understanding, that is, they had not noticed that odors were repeated and that the labels were manipulated. Thirteen participants (4 early blind, 2 late blind, and 7 sighted) had a partial understanding, as defined as having noticed that one or several odors were repeated, but not that odors were presented under different labels. Twenty-one participants (eight early blind, four late blind, and nine sighted) had full understanding, that is, had noticed that one or several odors were repeated under different labels. To investigate whether the level of understanding had any substantial influence on the olfactory illusions, separate ANOVAs were performed for participants with partial or full understanding (*n* = 34). The main effects of odor label on pleasantness and familiarity ratings remained statistically significant (*p*s < .001). Specifically, the main effect of odor label, *F*(2, 62) = 51.87, *p* < .001, η^2^ = .56, and the interaction between odor label and visual status, *F*(4, 62) = 4.86, *p* = .002, η^2^ = .10, for pleasantness ratings remained statistically significant. Likewise, the main effect of odor label on familiarity ratings remained statistically significant, *F*(2, 62) = 9.77, *p* < .001, η^2^ = .23. However, the influence of odor label, *F*(2, 62) = 0.72, *p* = .49, η^2^ = .02, and visual status, *F*(2, 31) = 2.79, *p* = .08, η^2^ = .15, on intensity ratings did not remain statistically significant. Taken together, this indicated that the olfactory valence illusion was not a function of demand characteristics and that odors were rated according to the valence of the label regardless of the participants’ understanding of the experimental design.

### Associations Between Perceptual Ratings

Collapsed across study groups, there was a positive correlation between pleasantness and familiarity ratings, and this was true for odors with negative labels (*r*_s_ = .41, *p* = .001), neutral labels (*r*_s_ = .48, *p* < .001), and positive labels (*r*_s_ = .35, *p* = .005).

### Odor Identification

Odor identification performance was similar for early blind (*M* = 12.7, *SD* = 2.2), late blind (*M* = 13.1, *SD* = 1.9), and sighted participants (*M* = 12.4, *SD* = 2.2), *F*(2, 61) = 0.70, *p* = .50, η^2^ = .02.

## Discussion

In this study, we addressed if visual processing in the form of visual imagery modulates verbally induced olfactory illusions by studying early and late blind participants. We hypothesized that early blind participants would be less affected by the valence of odor labels when judging the pleasantness of the odors compared with sighted and late blind individuals. However, the results indicated the opposite pattern as blindness, both early and late, was related to stronger olfactory illusions—at least in terms of pleasantness evaluations. This clearly demonstrates that visual imagery did not play a role in how odors were perceived and suggests that blind participants attended to the auditory verbal information more than the sighted.

Increased multisensory illusions in blind participants contrasts earlier findings showing that blindness induces a reduction in multisensory illusions—a finding that has been attributed to weaker multisensory integration (Hötting & Röder, 2004, 2009; Occelli et al., 2013). Enhanced perceptual skills (e.g., increased pitch discrimination and sound localization) have been put forward as the mechanism for weaker multisensory integration in these senses (Hötting & Röder, 2009). The principle of inverse effectiveness states that the likelihood of multisensory integration is greatest in noisy environments or when the input of each single modality is weak (Holmes, 2009; Stein & Meredith, 1993). Thus, as blind individuals have better auditory and tactile perceptual skills the likelihood of multisensory integration is also lower (Hötting & Röder, 2009). However, it should be noted that all previous studies on multisensory illusions in congenitally blind individuals have focused on perceptual tasks in the auditory and tactile senses, where increases in perceptual abilities have been demonstrated (Hötting & Röder, 2004, 2009). In contrast, the majority of olfactory studies demonstrate that olfactory abilities are similar for blind and sighted (Cornell Kärnekull et al., 2016, 2018, 2020; Sorokowska et al., 2019). Thus, based on this fact and earlier multisensory illusions, blind individuals should be as susceptible to multisensory odor illusions as sighted.

Whereas our result could be taken as an indication that enhanced perceptual skills may be a prerequisite for weaker multisensory illusions, it does not explain why the illusion was stronger in blind participants. This indicates that other mechanisms must be involved. One possibility is that blind individuals rely more upon verbal descriptions when interpreting their surroundings. This can make it intrinsically harder to separate the semantic knowledge attached to an odor label from the odor percept per se.

In line with our hypothesis and previous research, odors that were associated with positive labels were perceived as more pleasant than those associated with neutral or negative labels, and those with neutral labels were perceived as more pleasant than those with negative labels (de Araujo et al., 2005; Djordjevic et al., 2008; Herz & von Clef, 2001; Manescu et al., 2014). This pattern was observed in early blind, late blind, and sighted participants, respectively. Olfactory illusions of perceived pleasantness have previously been associated with increased activity in secondary olfactory cortex (i.e., medial orbitofrontal cortex [OFC], adjoining anterior cingulate cortex [ACC], and amygdala), and pleasantness ratings has been shown to correlate positively with the magnitude of the activation in OFC and ACC (de Araujo et al., 2005). These findings have been interpreted as evidence of modulations in olfactory processing of pleasantness per se and not only as a cognitive bias on the ratings (de Araujo et al., 2005).

Interestingly, de Araujo et al. also showed that the same olfactory brain regions, in particular the OFC and ACC, can be influenced by the valence of the labels even when there is no odor present, which suggests that participants may imagine the odors. This is further supported by the fact that several studies have demonstrated that verbal labels of odors can evoke activity in primary and secondary olfactory areas (Arshamian et al., 2013; Arshamian & Larsson, 2014; Flohr et al., 2014; González et al., 2006). This is important to consider when regarding the potential role played by imagery as previous olfactory illusion studies have suggested that colors may evoke mental visual images of the odor sources and thereby affect perception (Morrot et al., 2001). In our study, however, participants were explicitly cued by the odor label and our results show that visual imagery did not play a role. This opens the question of whether enhanced imagery in the remaining sensory modalities (e.g., auditory and tactile) might have played a role in why early and late blind individuals were more affected by the olfactory illusion than were sighted. This would be in line with a previous study in our laboratory where we found that blind individuals, especially early blind, rated vividness of olfactory and auditory imagery as higher than sighted individuals (Cornell Kärnekull et al., 2016). For example, the odors may have evoked emotional auditory images related to the odors (e.g., in the form of autobiographical memories) more strongly in the blind participants. Moreover, the strength of the olfactory illusion might have been modulated by group differences in sniffing behavior, as sniffing per se has been shown to influence odor imagery vividness—an effect especially pronounced in participants with reported good odor imagery (Arshamian et al., 2008; Bensafi et al., 2003, 2005).

Our hypothesis that the intensity ratings of early blind individuals would be less susceptible to the olfactory illusion than late blind and sighted individuals was not supported. However, we found a small effect of label on intensity ratings in terms of higher ratings of intensity for odors with positive labels than for those with neutral and negative labels, with only the difference between positive and neutral labels being statistically significant. Hence, the hypothesis that negative odor labels would result in higher perceived odor intensity than neutral and positive labels was also not supported.

There was an overall group difference in intensity ratings, with increasing ratings from sighted to late blind to early blind participants, with a statistically significant difference between the early blind and sighted. The reason for these group differences is unclear, as most research indicates that basic olfactory functions such as detection thresholds are similar for blind and sighted individuals (Cornell Kärnekull et al., 2016; Luers et al., 2014; Smith et al., 1993; Sorokowska, 2016; Sorokowska et al., 2019). It should be noted that whereas the effect of label on odor pleasantness appears to be robust, the influence on other perceptual dimensions, such as intensity, has varied between studies (de Araujo et al., 2005; Djordjevic et al., 2008; Herz & von Clef, 2001; Manescu et al., 2014). In previous research, most studies have reported higher intensity for odors associated with negative labels as compared with positive labels (Djordjevic et al., 2008; Herz & von Clef, 2001), whereas others have either not observed any statistically significant difference in ratings (de Araujo et al., 2005) or have found higher ratings for positive than negative labels (Manescu et al., 2014).

Previous mixed findings could result from methodological differences between the studies. For example, some studies only used negative and positive labels (e.g., de Araujo et al., 2005; Herz & von Clef, 2001; Manescu et al., 2014), whereas Djordjevic et al. (2008) also included neutral labels. Also, while Herz and von Clef (2001) tested participants on two occasions with different labels each time, others presented the same odors with different labels at one occasion (i.e., de Araujo et al., 2005; Djordjevic et al., 2008; Manescu et al., 2014). Moreover, the large differences between studies in the number of odors used and number of participants tested (12–80) could partially explain the discrepancies as these factors directly affect both the validity and the statistical power of the studies.

Finally, perceived odor familiarity was also affected by the valence of the labels, with higher ratings for positive than neutral and negative labels, and a statistically significant difference between positive and negative labels. We had no preregistered hypotheses regarding olfactory illusions in perceived familiarity as we are aware of only one previous study addressing this question (Herz & von Clef, 2001). Our findings, however, are in line with the study by Herz and von Clef (2001), in which most odors with positive labels were rated as more familiar than those with negative labels. The largest and only statistically significant effect in that study was observed for a mix of isovaleric and butyric acids. We did not find any overall differences in familiarity ratings between early blind, late blind, and sighted individuals, and the effect of label did not interact with this factor. In addition, in line with our hypothesis and previous research, we observed a positive correlation between familiarity and pleasantness ratings, irrespective of the valence of odor labels (Distel et al., 1999; Sulmont et al., 2002).

As the methodological paradigm was adapted from Djordjevic et al. (2008), odors were presented on a single occasion under different labels, and to minimize the risk that participants would notice that odors were repeated a number of distractor odors were interspersed with the experimental odors. Despite these precautions, a relatively large proportion of the participant sample had partial or full understanding of the study rationale (i.e., had noticed that one or several odors were repeated under different labels). However, based on a number of follow-up analyses, the main results and conclusions appear reliable and robust. Hence, even when separately analyzing the data for participants with partial or full understanding of the study, the results of the pleasantness and familiarity ratings remained the same. The effect of odor label on intensity ratings was small before stratifying the data and the fact that this effect was no longer statistically significant in the control analyses might have been due to low statistical power.

While this study eliminates visual imagery as a potential driver of verbally induced olfactory illusions, the unexpected finding that blind individuals had stronger illusions indicates that the mechanism behind this illusion must depend on one or several factors that are modulated by blindness. One factor could be the documented increase in vividness of olfactory and auditory mental images (Cornell Kärnekull et al., 2016). Future studies targeting olfactory illusions (in both in sighted and blind) should ask participants if the odor labels evoke mental images or episodic memories and have them rate their vividness so as to test if the strength of odor illusions is partially a function of mental imagery.

## Conclusions

Here we have shown that visual information (e.g., in the form of visual imagery or multisensory integration between visual imagery and perception) does not contribute to verbally induced olfactory illusions. Contrary to our hypotheses, that blindness would reduce the influence of verbal information on olfactory perception, the illusion was in fact amplified. Hence, pleasantness ratings of early and late blind individuals were more affected by odor names than that of sighted, and the effect size was quite substantial. Taken together, our results indicate that the general mechanism underlying verbally induced olfactory illusions must be found elsewhere than in visual processing per se. As for the reason why blind participants showed stronger illusions than sighted, this could potentially be due to more reliance upon the verbal context for blind individuals than the sighted and differential mental imagery capacity in other modalities (e.g., olfaction, audition, and touch).
